# The Role of Metal Components in the Cardiovascular Effects of PM_2.5_

**DOI:** 10.1371/journal.pone.0083782

**Published:** 2013-12-27

**Authors:** Jingping Niu, Eric N. Liberda, Song Qu, Xinbiao Guo, Xiaomei Li, Jingjing Zhang, Junliang Meng, Bing Yan, Nairong Li, Mianhua Zhong, Kazuhiko Ito, Rachel Wildman, Hong Liu, Lung Chi Chen, Qingshan Qu

**Affiliations:** 1 Lanzhou University School of Public Health, Lanzhou, China; 2 Nelson Institute of Environmental Medicine, New York University School of Medicine, Tuxedo, New York, United States of America; 3 New York University College of Arts and Sciences, New York City, New York, United States of America; 4 Peking University School of Public Health, Beijing, China;; 5 Department of Epidemiology and Population Health, Albert Einstein College of Medicine, Bronx, New York, United States of America; Max-Delbrück Center for Molecular Medicine (MDC), Germany

## Abstract

Exposure to ambient fine particulate matter (PM_2.5_) increases risks for cardiovascular disorders (CVD). However, the mechanisms and components responsible for the effects are poorly understood. Based on our previous murine exposure studies, a translational pilot study was conducted in female residents of Jinchang and Zhangye, China, to test the hypothesis that specific chemical component of PM_2.5_ is responsible for PM_2.5_ associated CVD. Daily ambient and personal exposures to PM_2.5_ and 35 elements were measured in the two cities. A total of 60 healthy nonsmoking adult women residents were recruited for measurements of inflammation biomarkers. In addition, circulating endothelial progenitor cells (CEPCs) were also measured in 20 subjects. The ambient levels of PM_2.5_ were comparable between Jinchang and Zhangye (47.4 and 54.5µg/m^3^, respectively). However, the levels of nickel, copper, arsenic, and selenium in Jinchang were 82, 26, 12, and 6 fold higher than Zhangye, respectively. The levels of C-reactive protein (3.44±3.46 vs. 1.55±1.13), interleukin-6 (1.65±1.17 vs. 1.09±0.60), and vascular endothelial growth factor (117.6±217.0 vs. 22.7±21.3) were significantly higher in Jinchang. Furthermore, all phenotypes of CEPCs were significantly lower in subjects recruited from Jinchang than those from Zhangye. These results suggest that specific metals may be important components responsible for PM_2.5_-induced cardiovascular effects and that the reduced capacity of endothelial repair may play a critical role.

## Introduction

Ambient air particulate matter (PM) is a heterogeneous mixture that varies in particle size and chemical composition. Many studies have demonstrated a consistent increased risk for cardiovascular disorders (CVD) in humans with either short- or long-term exposures to PM less than 2.5µm (PM_2.5_) at levels currently encountered in the U.S. [1]. However, the underlying mechanism(s) and major component(s) responsible for PM_2.5_ associated CVD are still poorly understood. In our recent mouse inhalation studies [2-5], we have found that exposure to inhaled nickel nanoparticles, or of PM_2.5_ with high nickel (Ni) content, caused alterations in heart rate (HR) and heart rate variability (HRV), altered vasoreactivity, induced adverse effects in bone marrow endothelial progenitor cells and advanced the progression of atherosclerosis. Human epidemiological data related to PM exposure [6-9] and other emerging animal research [8-10], has shown results similar to our group’s findings. Thus, we hypothesized that Ni or other specific metals, although a minor mass component, plays a critical role in PM_2.5_ induced CVD (for a full review of PM_2.5_ including its composition and hypothesized mechanisms of cardiovascular disease, please refer to the US EPA Air Quality Criteria for Particulate Matter (October 2004) Volume I and II reports; http://cfpub2.epa.gov/ncea/cfm/recordisplay.cfm?deid=87903). It has been difficult, however, to test this hypothesis in humans due to the lack of appropriate exposure environment, populations, and useful biological endpoints that may be employed.

Recently, we identified two cities in China, Jinchang and Zhangye, which have similar levels of PM_2.5_, but very different concentrations in Ni, copper (Cu), arsenic (As), and selenium (Se). These cities provided an ideal environmental setting to not only further examine the role played by chemical components in PM_2.5_-associated CVD, but also explore its potential mechanisms, as we further hypothesized that inflammation, as well as endothelial damage and impairment of subsequent repair are responsible for the specific component-associated adverse cardiovascular effects of PM_2.5_ exposure. Circulating endothelial progenitor cells (CEPCs) have been successfully used as biomarkers of disrupted endothelium integrity [11]. Their numbers in peripheral blood have also been correlated with cardiovascular disease risk [12-14], which make it possible to evaluate whether endothelial damage and impairment of subsequent repair are underlying mechanisms of PM_2.5_-associated CVD. Thus, the present study tested these hypotheses by examining various markers of cardiac and vascular integrity, inflammation, and disrupted endothelial repair in human subjects recruited from both cities.

## Methods

### Study Sites and Subject Recruitment

This study was conducted in two large adjacent communities in Gansu Province, China: Jinchang (JC) and Zhangye (ZH), with comparable ambient concentrations of PM_2.5_. Jinchang was identified as a heavily Ni-polluted area due to its proximity to the second largest Ni refinery in the world. Zhangye, 250 miles northwest and upwind from Jinchang, was selected to serve as a control community. The human subject protocol for this study was approved by the IRBs of both the New York University School of Medicine and the Peking University Health Science Center. Written informed consent was obtained from all participating subjects. 

For subject recruitment, we first identified a small community in downtown area of Jinchang within 0.5 miles of the central ambient air monitoring site. The local residents at aged 60 to 65 (susceptible subpopulation to PM_2.5_ associated CVD) were first targeted as a population pool for recruitment. Males were excluded from this exploratory study because it was difficult to find non-smoking male subjects in these communities. Therefore, the recruitment procedures, included a questionnaire interview, physical examination, and lab tests, and were only conducted among the local elderly non-smoking female residents. During the interviews, age, BMI, and blood pressure levels were recorded. Subjects with abnormal blood sugar and lipid profiles and who had diagnosed diseases, including CVD, diabetes, and hypertension, were excluded from enrollment. Finally, 30 nonsmoking and healthy female subjects with age between 60 and 65 were randomly selected among 45 and 46 residents in Jinchang and Zhangye, respectively, and interviewed for this study. All participants were Han Chinese (95% of the residents in both cities are Han Chinese). With the same procedure, 30 nonsmoking and healthy female subjects were enrolled from Zhangye through individually matching them with subjects recruited in Jinchang by age, educational level, socioeconomic status, and lifestyle behaviors. The lifestyle factors matched for the two groups include ethnicity, smoking, alcohol consumption, diet, and physical activity. According to the current retirement policy in China, the mandatory retirement age for females is 55, and therefore, the study subjects were retired at least 5 years before the study began. Thus, matching for individual occupation was not considered. 

All 60 subjects provided blood samples for lipid profile, assays of CVD risk, and a smoking (cotinine) biomarker. Confirmation of non-smoking status and potential exposure to second-hand smoke were assessed by cotinine levels in plasma measured using ELISA kits (Calbiotech, Spring Valley, CA). In addition, a random subset of 10 subjects in each city (20 total) were selected from the 60 recruited subjects for measurements of CEPC, vascular endothelial growth factor (VEGF), and stromal cell-derived factor-1 (SDF-1α) analyses. 

### PM_2.5_ Sampling

Daily PM_2.5_ samples were collected on pre-weighed Teflon membrane filters (Teflo, Pall, NY) with PM_2.5_ sharp-cut cyclone inlets, simultaneously in the downtown areas of both Jinchang and Zhangye for an entire period of 12 months. The filters were retrieved every 24 hrs, and post-weighed to determine PM_2.5_ mass. Analyses for 35 elements were done by non-destructive X-ray fluorescence spectroscopy (XRF) (Jordan Valley EX-6600 –AF, Austin, TX) using five secondary fluorescers (Si, Ti, Fe, Ge, and Mo), and spectral software XRF2000v3.1 (U.S. EPA and ManTech Environmental Technology, Inc.) according to the method described in our previous study [15]. In addition, personal sampling of PM_2.5_ mass concentrations and of its 35 chemical components was also conducted for the 60 subjects, by use of a backpack containing a personal pump connected to a cyclone and filter cassette with a pre-weighed Teflon filter to collect PM_2.5_ samples for 24 hours, on the days when blood samples were collected for inflammatory biomarkers analyses according to the method described in our previous study [16].

### CVD Risk Biomarkers

A total of 20 ml of blood was collected in two heparin containing vacutainer tubes from each subject by a local registered nurse at the end of personal exposure monitoring. One 10 ml tube of whole blood was spun down at 300g for 15 minutes to separate plasma for analyses of vascular endothelial growth factor (VEGF; RND Systems, Minneapolis, MN), stromal cell-derived factor-1 (SDF-1α; RND Systems, Minneapolis, MN), C-reactive protein (CRP; Bender Medsystems, San Diego, CA), interleukin-6 (IL-6; Bender Medsystems, San Diego, CA), inter-cellular adhesion molecule-1 (ICAM-1; Bender Medsystems, San Diego, CA), monocyte chemotactic protein-1 (MCP-1; Bender Medsystems, San Diego, CA) and vascular cell adhesion molecule-1 (VCAM-1; Bender Medsystems, San Diego, CA). All assays were conducted in duplicate with corresponding ELISA kits according to the manufactures’ instructions. The remaining 10ml tube of blood was used for flow cytometry measurement of CEPCs (see below). 

### Circulating Endothelial Progenitor Cells (CEPCs), VEGF, and SDF-1α

CEPCs falling within lympho-mononuclear cell populations were discriminated from each other based on the expression of 3 groups of cell surface markers using flow cytometry. Three phenotypes were chosen due to the lack of consensus of an EPC surface marker definition. CEPCs were enumerated using cells labeled with CD34+/KDR+, CD34+/KDR+/CD45-, or CD34+/KDR+/CD133+. Aliquots of blood samples (200 µl) were processed by lysing the red blood cells using FACS lysis solution (BD Biosciences, San Jose, CA). Non-specific antibody binding was blocked using 20 µl of specific Fc-receptor antibodies (Miltenyi Biotec, Auburn, CA) for 20 min at room temperature. Cells were then incubated in the dark with fluorochrome-labelled anti-human antibodies FITC-CD34 (BD Biosciences, San Jose, CA), PE-KDR (BD Biosciences, San Jose, CA) and/or Percp-CD45 (BD Biosciences, San Jose, CA) or Percp-CD133 (eBioscience, San Diego, CA) for 30 min at 4°C. Corresponding isotypes from mouse immunoglobulin IgG1 were used as controls. Flow cytometry was performed on a BD FACSCalibur (BD Biosciences, San Jose, CA) using forward and side scatter (FSC/SSC) instrument settings and gated to include only lympho-mononuclear cell events. A second gate was used to include only those cells negative for CD45 or positive for CD133. A final gate was used to analyze cells positive for both CD34 and KDR expression (See Figure S1). A minimum of 300,000 events were recorded per sample. Measurements were conducted in plasma for VEGF and SDF-1α according to the standard procedures provided by the manufacturer (RND Systems, Mineapolis, MN). 

### Statistical Analysis

We conducted an examination of the difference in inflammatory biomarkers in subject living in the two cities (30 subjects in each city) as a function of the levels of personal exposures to PM_2.5_ and its chemical components, adjusting for individual risk factors. 

Pearson correlation analysis was employed to describe the relationships between endothelial related alterations (CEPCs, ICAM-1, VCAM-1, VEGF, and SDF-1α) and proinflammatory changes (CRP, MCP-1 and IL-6). Log transformation was performed on CRP, IL-6, and VCAM-1 to improve normality for statistical models. Note that, while we describe the difference in PM_2.5_ and its elemental compositions between the two cities, this part of analysis cannot distinguish the roles played by the individual trace element because the comparison is dichotomous (i.e., one degree of freedom) for the pollutants.

In the analysis of inflammatory markers, the effects of air pollutants can be either short-term or long-term. Therefore, for this analysis, we used the personal exposure measurements taken on the day of biomarker measurements, which may reflect the concentrations of the pollutant at both the time and location of the subject on that day (though the generally higher levels of the trace elements in Jinchang may still dominate the contrast in concentrations). We chose Ni, copper (Cu), arsenic (As), and selenium (Se) as the key element of interest for the analysis since they were present in highest concentrations in the Ni-smelter city (JC) compared to the control city (ZH). The personal exposure levels of these elements across the subjects were highly skewed, and therefore, we used log-transformed values for analysis. We first examined the associations of the concentrations of these pollutants and individual level risk factors (e.g., age, BMI, cotinine level, lipid profile, and blood pressure levels), expressed as a percent increase from the average of the inflammatory biomarker per an inter-quartile-range change in the pollution or risk factor variable in univariate regression models. Then, we estimated percent increase the pollution variables simultaneously adjusting for all of the personal-level risk factors in multivariate regression models. Correlations between elements in Jinchang and Zhangye are presented in Figure S2 and S3. 

Statistical analyses were conducted in R (v.2.12.2) for the student’s *t* test, Pearson correlation analysis and multiple linear regression models. Statistical significance for all analyses was determined using a two-sided alpha of 0.05. 

## Results

### Ambient concentrations of PM_2.5_ and 35 elements

**Figure 1:**
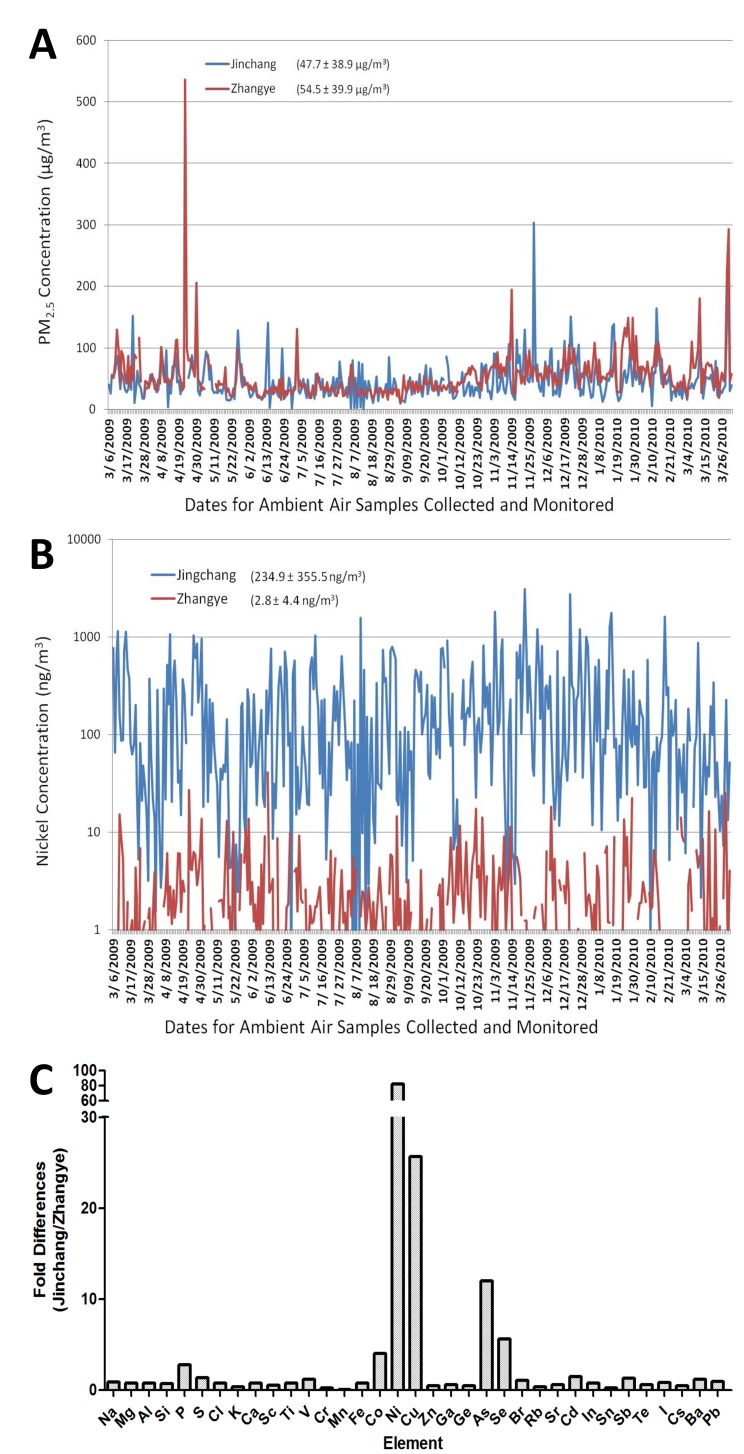
Differences in daily ambient exposures between Jinchang and Zhangye from March 6^th^ to April 4^th^, 2010. Panel A: PM_2.5_ Mass Concentrations; Panel B: Nickel Mass Concentrations; Panel C: Fold differences in concentrations of 35 elements between Jinchang over Zhangye.

The measurements of PM_2.5_ and elements were completed for air samples collected between March 6^th^ 2009 and April 4^th^, 2010. The daily variations of PM_2.5_, Ni, and fold differences in all elements are shown in Figure 1(A, B, C). No significant difference in PM_2.5_ mass was detected between Jinchang (47.4±38.9 µg/m^3^) and Zhangye (54.5±39.8 µg/m^3^) (Figure 1a) and the levels of PM_2.5_ in both cities were approximately twice as high as the annual averages seen in New York City (20.2±13.3 µg/m^3^) [17]. However, the ambient level of Ni in Jinchang (234.5±354.3 ng/m^3^) was 82-fold higher than that in Zhangye (2.8±4.4 ng/m^3^, which is comparable to the ambient level of Ni in Newhaven, CT (Figure 1b) [18]. In addition to Ni, mean levels of Cu, As, and Se were 26, 12, and 6 fold higher in Jinchang than in Zhangye, respectively (Figure 1c). Table 1 shows a correlation matrix of personal exposure PM_2.5_ and the key elements with and without log-transformation. PM_2.5_ is poorly correlated with the trace metals, either with or without log-transformation. Cu, As, and Se are more highly correlated with each other in raw data than in log-transformed data, whereas Ni’s correlations with Cu and Se improved when data were log-transformed, indicating the influence of extreme values. These results indicated that Cu, As, and Se were derived from the same source, most likely produced by coal combustion with As and Se as signature elements, while Ni component of PM_2.5_ was from a separate source, i.e. the nickel refinery. Table 2 shows the correlations between personal exposure and central monitoring sites. Similar to that described by others ^50^, it is not surprising to observe that PM_2.5_ correlates poorly between personal exposure and central site in Jinchang. However, other key elements have excellent correlations between personal and central site concentrations, implying that exposures to the two main sources (Ni refinery and coal power plant) were fairly uniform in Jinchang and data of central site monitor can be used to represent the personal exposure levels. In contrast, in Zhangye, Cu and As have good correlations between personal exposure and central site measurement while PM_2.5_, Ni and Se did not. One possible reason for this lack of correlation was that the concentrations of these elements were much lower in Zhangye and some measurements were near the detection limits of our XRF system.

**Table 1:** Correlation Matrix of Personal Exposures to PM_2.5_ and key metal concentrations (n=57).

	**PM_2.5_**	**Ni**	**Cu**	**As**	**Se**
**PM_2.5_**	1	0.11	0.15	0.14	0.29
**Ni**	0.12	1	0.8	0.57	0.64
**Cu**	0.17	0.56	1	0.71	0.7
**As**	0.25	0.59	0.94	1	0.7
**Se**	0.28	0.49	0.88	0.86	1

The numbers in the upper triangle are correlations using log-transformed metals concentrations. The numbers in the lower triangle are correlations using raw data.

**Table 2:** Correlations of personal exposure and central site concentrations of PM_2.5_ and key elements in Jingchang and Zhangye.

	**PM_2.5_**	**Ni**	**Cu**	**As**	**Se**
**Jingchang**	0.05	0.42	0.74	0.8	0.52
**Zhangye**	0.16	0.12	0.54	0.32	-0.15

### Changes in CVD risk markers

As shown in Table 3, no significant differences were detected between subjects recruited from the two cities for age, BMI, lipid profiles, blood pressure, or blood sugar. All CVD risk biomarkers for the 60 individuals, except ICAM-1, were elevated in subjects recruited from Jinchang compared with those from Zhangye, while significant differences were only detected for inflammatory markers CRP and IL-6 (p<0.01 and 0.05, respectively). 

**Table 3:** Characteristics of all 60 Subjects, Environmental Exposure, and CVD Biomarkers (+SD).

	**Jinchang**	**Zhangye**	**P values**
**Age**	62.5 ± 2.1	62.0 ± 1.9	>0.05
**BMI**	23.2 ± 2.5	23.0 ± 2.2	>0.05
**Blood sugar** (**mmol/L**)	5.23 ± 0.60	5.60 ± 0.76	>0.05
**Blood Pressure** (**mmHg**)			
Systolic	129.5 ± 14.1	120.7 ± 10.8	>0.05
Diastolic	80.1 ± 8.0	77.7 ± 7.8	>0.05
**Cholesterol** (**mmol/L**)			
Total	4.80 ± 0.81	4.84 ± 0.84	>0.05
Low-density lipoprotein	2.50 ± 0.67	2.46 ± 0.45	>0.05
High-density lipoprotein	1.52 ± 0.29	1.62 ± 0.39	>0.05
Triglycerides	1.69 ± 0.36	1.65 ± 0.40	>0.05
**Environmental Exposure**			
Ambient levels of PM_2.5_ (µg/m^3^)	47.4±38.9	54.5±39.8	>0.05
Ambient levels of Ni (ng/m^3^)	234.5±354.3	2.8±4.4	<0.0001
Ambient levels of As (ng/m^3^)	143.2±164.4	11.9±11.3	<0.0001
Ambient levels of Se (ng/m^3^)	19.4±64.3	3.5±3.5	<0.0001
Ambient levels of Cu (ng/m^3^)	121.3±129.2	4.7±5.7	<0.0001
**CVD Biomarkers**			
CRP (mg/L)	3.44 ± 3.46	1.55 ± 1.13	0.0017**^*҂*^**
IL-6 (pg/ml)	1.65 ± 1.17	1.09 ± 0.60	0.0433**^*҂*^**
MCP-1 (pg/ml)	462.61 ± 305.95	394.58 ± 102.73	>0.05
ICAM-1 (ng/ml)	272.81 ± 91.12	323.0 ± 128.77	>0.05
VCAM-1 (ng/ml)	1086.33 ± 451.69	920.88 ± 255.13	>0.05**^*҂*^**
**Central Ambient Exposure Monitoring**			
PM_2.5_ (µg/m^3^)	43.0±40.7	45.5±47.7	>0.05
Ni (ng/m^3^)	204.8±268.6	2.7±4.3	<0.0001
As (ng/m^3^)	101.64 ± 120.22	5.99 ± 8.78	<0.0001
Se (ng/m^3^)	10.53 ± 11.93	1.47 ± 1.39	<0.0001
Cu (ng/m^3^)	104.42 ± 117.22	4.17 ± 4.28	<0.0001
**Personal Exposure Monitoring**			
PM_2.5_ (µg/m^3^)	62.45±45.24	70.62±46.69	>0.05
Ni (ng/m^3^)	71.28±98.12	4.88±10.02	0.0060
As (ng/m^3^)	46.30 ± 50.52	9.00 ± 8.69	0.0002
Se (ng/m^3^)	7.92 ± 11.73	1.10 ± 1.66	0.0031
Cu (ng/m^3^)	61.21 ± 85.65	3.08 ± 3.63	0.0006

^҂^ Datum was log normalized for statistical analyses

Of the 60 subjects, only six subjects (three in each city) had detectable plasma cotinine levels, with no indication that one city had higher levels than the other (12.4 ± 22.5 ng/mL in Jinchang; 14.1 ± 44.5 ng/mL in Zhangye). However, based on a review of cotinine levels in smoking and non-smoking populations[19], the levels above 2 ng/mL indicated exposures to potential second-hand smoke or potential active smoking. Therefore, we adjusted for cotinine levels using an indicator variable (1 for the subjects whose cotinine levels were above 2 ng/mL) in the multivariate regression model of the CVD markers.

**Figure 2:**
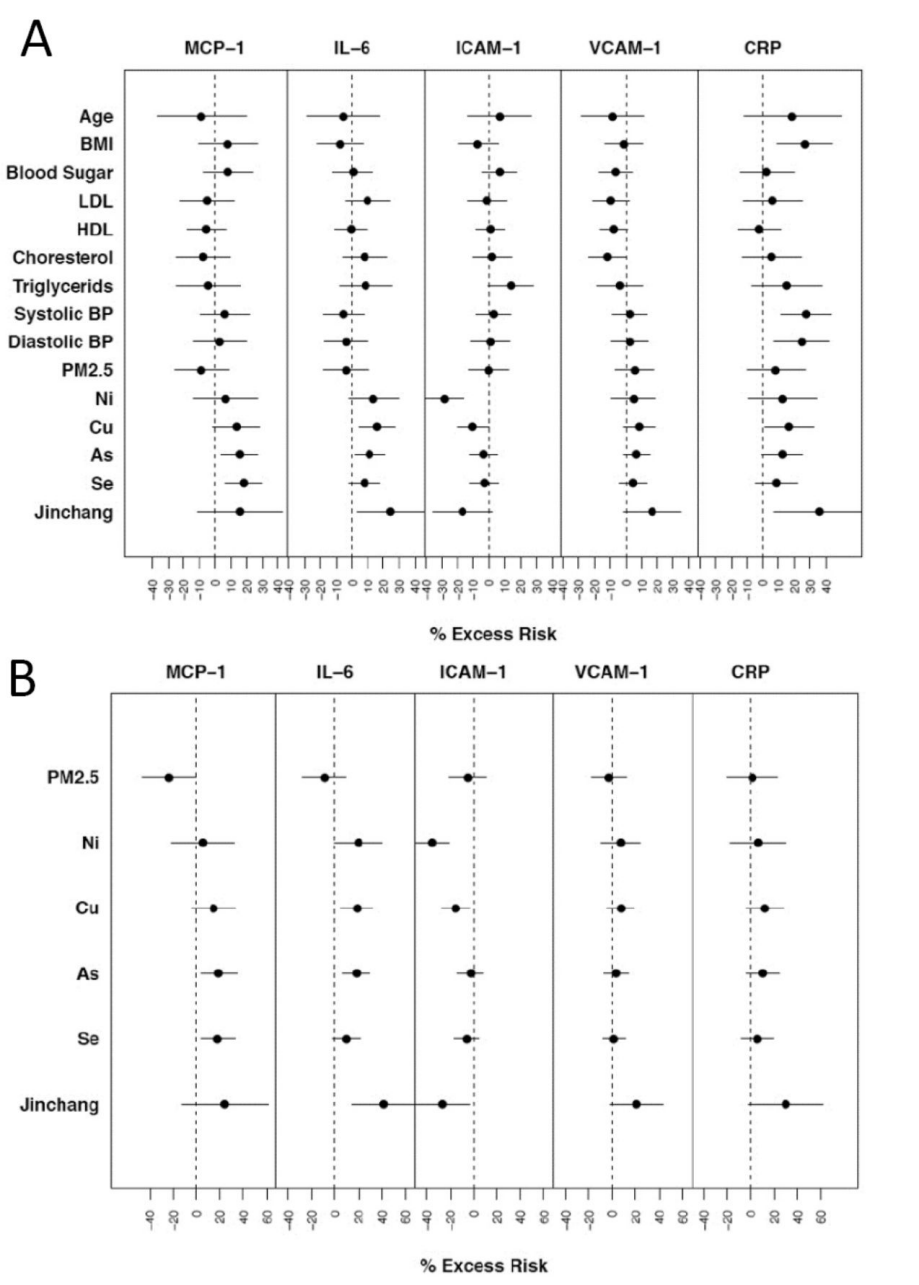
Percent excess risk from the mean values of biomarkers per inter-quartile-range increase in : 1) risk factors and air pollution in univariate regression model. “Jinchang” is an indicator (Jinchang=1; Zhangye=0). Metal concentrations are log-transformed (Panel A); 2) PM2.5 and selected metals, adjusting for age, cotinine level, BMI, blood sugar, LDL, HDL, triglycerides, systolic and diastolic blood pressure. Metal concentrations are log-transformed (Panel B).

When the CVD risk biomarkers were examined for their associations with the pollutants and risk factors individually, several notable patterns were observed (Figure 2a). Consistent with our expectation, BMI, systolic, and diastolic blood pressures were significantly positively associated with CRP. Cotinine level was also positively (though not significantly) associated with CRP, IL-6, ICAM-1, and (significantly) with VCAM-1. Residing in Jinchang was generally more significantly associated with CRP than personal (one day) exposure variables were. Residence in Jinchang and personal exposures to Cu were significantly positively associated (less so for other metals) with IL-6, but none of the individual-level risk factors were associated with this biomarker in the bivariate regression models. The personal exposure levels to As and Se were more strongly positively associated with MCP-1 than with living in Jinchang. Ni was significantly negatively associated with ICAM-1, but living in Jinchang was also nearly significantly negatively associated with this biomarker. VCAM-1 was nearly significantly positively associated with living in Jinchang. To conservatively estimate pollution effects, we conducted multivariate regression analysis with all the individual-level risk factors included (Figure 2b). Se and As remained significant predictors of MCP-1. Cu and As remained significant predictors of IL-6, though not as significantly as the Jinchang indicator variable. Ni, Cu, and the Jinchang indicator variable were negatively significantly associated with ICAM-1. Overall, except for ICAM-1, the personal exposure levels to these metals and living in Jinchang, but not PM_2.5_, were positively associated with inflammatory biomarkers. Re-analyzing the data without the six subjects with measurable levels of cotinine did not change the pattern of associations between the pollutants and the biomarkers described above (not shown). 

### Alterations of CEPCs, VEGF, and SDF-1α

The flow cytometry analyses in the 20-subject subset showed that the numbers of CEPCs, regardless of which phenotypic definition was used, were significantly lower in subjects from Jinchang compared to those from Zhangye (Table 4). Furthermore, within the 20 subjects, significant differences were found between locations for plasma VEGF but not for SDF-1α. However, Pearson correlation analysis for SDF-1α showed significant association with CRP (Table 5). Four out of the 20 subjects (3 in Jinchang and 1 in Zhangye) had measurable levels of cotinine. 

**Table 4:** Characteristics of 20 Subjects, CVD Biomarkers, CEPC Count, and Vascular Injury (+SD). ***^҂^***.

		**Jinchang**	**Zhangye**	**P values**
**Age**		61.9± 2.1	61.9 ± 1.9	>0.05
**BMI**		23.2 ± 1.8	22.9.0 ± 3.0	>0.05
**Blood sugar** (**mmol/L**)		5.32 ± 0.57	5.51 ± 0.54	>0.05
**Blood Pressure** (**mmHg**)				
Systolic		130.0 ± 12.3	122.0 ± 6.3	>0.05
Diastolic		79.0 ± 10.4	77.0 ± 6.8	>0.05
**Cholesterol** (**mmol/L**)				
Total		4.62 ± 0.78	4.42 ± 0.46	>0.05
Low-density lipoprotein		2.34 ± 0.61	2.33 ± 0.34	>0.05
High-density lipoprotein		1.53 ± 0.23	1.38 ± 0.22	>0.05
Triglycerides		1.63 ± 0.36	1.54 ± 0.43	>0.05
**CVD Biomarkers**				
CRP (mg/L)		4.78 ± 4.36	1.54 ± 1.12	0.05**^*҂*^**
IL-6 (pg/ml)		1.65 ± 1.26	0.90 ± 0.44	>0.05
MCP-1 (pg/ml)		570.76 ± 521.98	419.99 ± 66.77	>0.05
ICAM-1 (ng/ml)		275.95 ± 98.39	303.42 ± 62.0	>0.05
VCAM-1 (ng/ml)		1222.11 ± 468.84	903.25 ± 153.87	>0.05**^*҂*^**
**CEPCs** (**% of lymphocytes & monocytes**)				
CD34+/KDR+		0.049 ± 0.015	0.301 ± 0.155	0.0003
CD34+/KDR+/CD133+		0.013 ± 0.004	0.050 ± 0.037	0.0066
CD34+/KDR+/CD45-		0.003 ± 0.003	0.010 ± 0.006	0.0002
**Vascular Injury**				
VEGF (pg/ml)		117.6 ± 216.99	22.7 ± 21.35	0.0362**^*҂*^**
SDF-1α (pg/ml)		3186.9 ± 754.03	3384.2 ± 522.67	>0.05
**Personal Exposure Monitoring**				
PM_2.5_ (µg/m^3^)	80.42±51.91		77.22±47.08	>0.05
Ni (ng/m^3^)	72.74±87.32		3.82±3.22	0.0226
As (ng/m^3^)	67.70 ± 70.34		12.03 ± 10.52	0.0235
Se (ng/m^3^)	15.38 ± 17.08		1.49 ± 1.58	0.0197
Cu (ng/m^3^)	101.36 ± 129.23		3.99 ± 3.39	0.0285

Data was log normalized for statistical analyses

**Table 5:** Correlation Matrix of Biomarkers in Participating subjects*.

	**CD34+/KDR+**	**CD34+/KDR+/CD133+**	**CD34+/KDR+/CD45-**	**MCP-1**	**VCAM-1**	**ICAM-1**	**IL-6**	**CRP**	**VEGF**	**SDF-1α**
**CD34+/KDR+**	1									
**CD34+/KDR+/CD133+**	**0.9067 (<0.0001**)	1								
**CD34+/KDR+/CD45-**	**0.6329 (0.0027)**	**0.4709 (0.0361)**	1							
**MCP-1**	-0.102 (0.6662)	-0.077 (0.7463)	-0.262 (0.2636)	1						
**VCAM-1**	-0.345 (0.1352)	-0.280 (0.2309)	-0.319 (0.1702)	-0.048 (0.8402)	1					
**ICAM-1**	-0.073 (0.7595)	-0.134 (0.5705)	-0.196 (0.4056)	-0.095 (0.688)	0.3416 (0.1404)	1				
**IL-6**	-0.355 (0.1239)	-0.251 (0.2852)	-0.350 (0.1293)	0.0127 (0.9576)	0.3127 (0.1794)	-0.098 (0.6787)	1			
**CRP**	-0.373 (0.1051)	-0.233 (0.3215)	-0.316 (0.1741)	-0.137 (0.5629)	0.3566 (0.1227)	0.1723 (0.4674)	0.4281 (0.0596)	1		
**VEGF**	-0.250 (0.2862)	-0.181 (0.4429)	0.0010 (0.9966)	-0.054 (0.818)	0.1681 (0.4786)	-0.394 (0.0848)	-0.184 (0.436)	0.1629 (0.4924)	1	
**SDF-1α**	0.0634 (0.7904)	-0.142 (0.5501)	-0.062 (0.7927)	-0.0817 (0.9394)**	-0.087 (0.7144)	0.1308 (0.5824)	-0.290 (0.2147)	**-0.479 (0.0325**)	-0.147 (0.5346	1

*: p values are shown in parenthesis and red type denotes significant result. **: one outlier removed from analysis

## Discussion

In this study, the concentrations of ambient PM_2.5_ and its components were measured in two large adjacent communities in Gansu Province, China: Jinchang and Zhangye, over a one year period. Although their PM_2.5_ concentrations were similar, due to a local Ni refinery and possibly a coal plant, the levels of Ni, Cu, As, and Se in Jinchang were 82, 26, 12, and 6 fold higher than Zhangye, respectively. The contrast of the concentrations of these elements between these cities is ideal to test the hypothesis that specific components in PM_2.5_, although only a minor fraction of total PM_2.5_ mass, plays a critical role in PM_2.5_ induced CVD. After adjusting for individual risk factors using multivariate regression analysis, exposure to Cu, Se, As, and living in Jinchang, but not PM_2.5_, were positively associated with inflammatory biomarkers. CRP and IL-6 were found to be significantly higher in subjects in Jinchang than in those from Zhangye by 122% and 51%, respectively. In addition, Se, As, and Cu concentrations in PM_2.5_ were found to be significant predictors of MCP-1 and IL-6 levels.

Systemic inflammatory markers, such as CRP and IL-6, have been successfully used in cardiovascular research as risk markers [20]. A study with 88 elderly subjects [21] reported that a 100 µg/m^3^ increase in PM_2.5_ was associated with approximately a 0.81 mg/dL increase in CRP, which suggests that CRP is useful biomarker for effects of PM in CVD research. Our findings suggest that specific metal components of PM_2.5_ (Ni, Cu, Se, and As) may be responsible for the elevated levels of systemic inflammatory markers in Jinchang since these PM_2.5_ components have been known to induce ROS production and inflammation in both *in vitro* and *in vivo* studies [22-25]. 

As indicated in several recent reviews [26-29] both endothelial damage and repair are involved in the development of various types of CVD, especially in the early stage. However, the potential mechanism(s) of CVD were not well illustrated in human population research until the measurement of immunologically defined CEPCs in the peripheral blood became available and used for the assessment of cardiovascular risk [12,30]. CEPCs are derived from bone marrow and characterized as a subset of CD34^+^ hematopoietic stem cells co-expressing vascular endothelial growth factor receptor-2 (VEGFR-2/Flk-1/KDR), which are able to differentiate and replace the damaged endothelial cells [31]. As indicated in a recent review [28,32], under steady-state conditions, these progenitor cells are normally maintained in an undifferentiated and quiescent state, but are mobilized following physiological stress and subsequently home in on sites of vascular damage. However, their ability to participate in the processes of endothelial repair, remodeling, and functional improvement may be affected significantly by a number of cardiovascular risk factors and human exposures. For example, cigarette smoking is associated with a reduced number of CEPCs, together with an important impairment of CEPC differentiation and functional activities [33]. A more recent study [9] demonstrated that episodic exposure to PM_2.5_ induced decrease of CEPCs. Similar to these findings, our present study showed a significant decrease of CEPCs in subjects from Jinchang compared to those from Zhangye. Furthermore, VEGF, one of the two important proteins involved in the mobilization, homing, and differentiation of CEPCs, was found significantly higher in subjects from Jinchang than that in those from Zhangye. This increased VEGF is thought to be, at least partially, responsible for the decreased number of CEPCs since it enhances homing and adhesion of the mobilized CEPCs to the damaged vascular endothelial sites, which may further reduce the number of CEPCs stored in the bone marrow thereby potentially exhausting the pool [34,35].

In summary, the results of the present study clearly showed significant biomarker differences between subjects recruited from Jinchang and Zhangye in terms of CRP, IL-6, CEPCs, and VEGF. As indicated in Tables 3 and 4, the ambient levels of PM_2.5_ in Jinchang and Zhangye are very similar and the demographic characteristics and CVD risk factors are comparable between groups of subjects. Thus, it is reasonable to hypothesize that the variations in PM components may account for these differences in biological endpoints between Jinchang and Zhangye. Because Ni has the highest contrast in mean concentration between these cities it was our a priori hypothesis that Ni would be the most important component among those that were also elevated in Jinchang (Cu, As, and Se), to induce adverse CVD related changes. Ni has been shown to induce significant oxidative stress and inflammation in the pulmonary and extra-pulmonary organs and exacerbated the progression of atherosclerosis in a sensitive mouse model after 5-months of daily inhalation exposure [3]. Furthermore, Lippmann et al. [2] have previously shown that Ni concentrations that were similar to those encountered in this study caused heart rate variability alterations in exposed mice. Liberda et al. [4] have shown that inhaled Ni nanoparticles not only cause a reduction in number of bone marrow EPCs, but also a reduction in their function. Thus, these results are supportive, but do not provide conclusive evidence that Ni alone may play a critical role in PM_2.5_ associated CVD, since other elements were also found to be higher in Jinchang. 

In addition to Ni, As, Se and Cu were significantly higher in Jinchang compared to Zhangye. Arsenic exposure has been associated with a variety of adverse cardiovascular effects in humans [36,37] and its proposed mechanisms may be the same as for Ni. Furthermore, arsenic has been shown to exacerbate atherosclerotic plaque in mice [38], and result in ECG alterations linking to atherosclerosis [39] – a strong similarity to the effects of high levels of Ni in PM_2.5_ observed in Ni- exposed mice [2,15,17,40,41]. However, it is worth noting that most of these effects of As resulted from exposures to As in drinking water, and not through inhalation. A recent study by Xun [42] found no association between Se and atherosclerosis; however, high serum Se concentrations were associated with increased total and LDL cholesterol, a preclinical measurement of atherosclerotic risk [43]. Cu in mass adjusted PM_2.5_ exposure showed no significant association with microvascular function [44]. However, a case-control occupational exposure study from a Swedish copper smelter found that increased mortality cardiovascular diseases were associated with exposures to As, Cu, and Ni [45], although there were no accurate measurements of the exposure concentrations. The regression analyses conducted in the present study also showed associations, at various degrees, of CVD risk markers with Cu, As, and Se. However, it is difficult to separate individual roles of Ni, As, Cu and Se in affecting the inflammatory and cardiac parameters measured in this study because, first of all, they are highly correlated with each other (Table 1). Secondly, they share similar potentials and pathways to induce oxidative stress and inflammation responses. Therefore, it can be argued that the observed effects associated with Ni may have been due to the other metals present at elevated concentrations in Jinchang, or synergism among the elevated metal components in the PM (Figure 1c). Recent studies by Cahill [46] and Lippmann [2] found that reductions in Ni, vanadium (V) and sulfur dioxide (SO_2_), and not Cu, As, or Se, decreased cardiovascular and respiratory associated death. As, Cu and Se were present in negligible concentrations in California[46] and Northeastern US [2]. These elements, found in China at elevated concentrations, were not reduced during the intervention study [46] and did not affect cardiovascular or respiratory associated death. Thus, it is possible that the observed effects may have been due to Ni and not from As, Cu, or Se, or that these effects were due to a combination of Ni, As, Cu, and Se.

## Conclusions

Our study has shown that in a Chinese city with high levels of ambient Ni, As, Se, and Cu, and having a similar level of PM_2.5_, to another Chinese city without such elevated component levels, there were close associations of lower levels of reparative CEPCs. Additionally, the same exposures were associated with significantly higher plasma CRP and Il-6 levels.

The risk of cardiovascular health hazards due to PM_2.5_ exposure is a major public concern. Both short- and long-term exposure to PM_2.5_ are associated with increased risks of CVD related mortality and morbidity [2,47,48]. Furthermore, our previous study [2] has shown significant alterations in heart rate variability due to Ni (but not As, Se, or Cu) in PM_2.5_, an early warning of altered cardiovascular health. The results obtained from this study greatly enhance our understanding of the mechanism and the role of Ni, together with other metal and metalloid components, in PM associated CVD. Identifying a group of components (Ni, As, Cu, and Se) responsible for the increased risk is a major step toward establishing credible control strategy in reducing the exposure-related risk. The findings of this study not only bridge the current research gap in understanding the mechanisms of PM_2.5_- and metal-associated CVD, but also provide a new avenue for investigating cardiovascular effects of other toxic agents.

## Supporting Information

Figure S1 **CEPCs belonging to the mononuclear cell fraction, were identified using a lympho-monocyte (LM) gate by their scatter plots (**A**).** With LM gated, CD34+/KDR+ cells in the upper-right quadrant were quantified (B). Further gates included CD133+ (C) and CD45- cells in the mononuclear cell population, according to the corresponding isotype controls. Triple positive cells were identified in the CD133+ gate as cells positive for CD34 and KDR (C). Cells positive for both CD34 and KDR in CD45- gate as shown in the upper-right quadrant (D) were identified as CD34+/KDR+/CD45- .
(PNG)

Figure S2 **Correlation of Elements in Jinchang.** Correlations greater than 0.9 are presented in red.
(PNG)

Figure S3 **Correlation of Elements in Zhangye.** Correlations greater than 0.9 are presented in red.
(PNG)
