# Supplementary figures and images for: The Role of Metal Components in the Cardiovascular Effects of PM2.5
Source: PLoS One. 2013 Dec 27;8(12):e83782. doi: 10.1371/journal.pone.0083782 (PMC3873977; doi:10.1371/journal.pone.0083782)

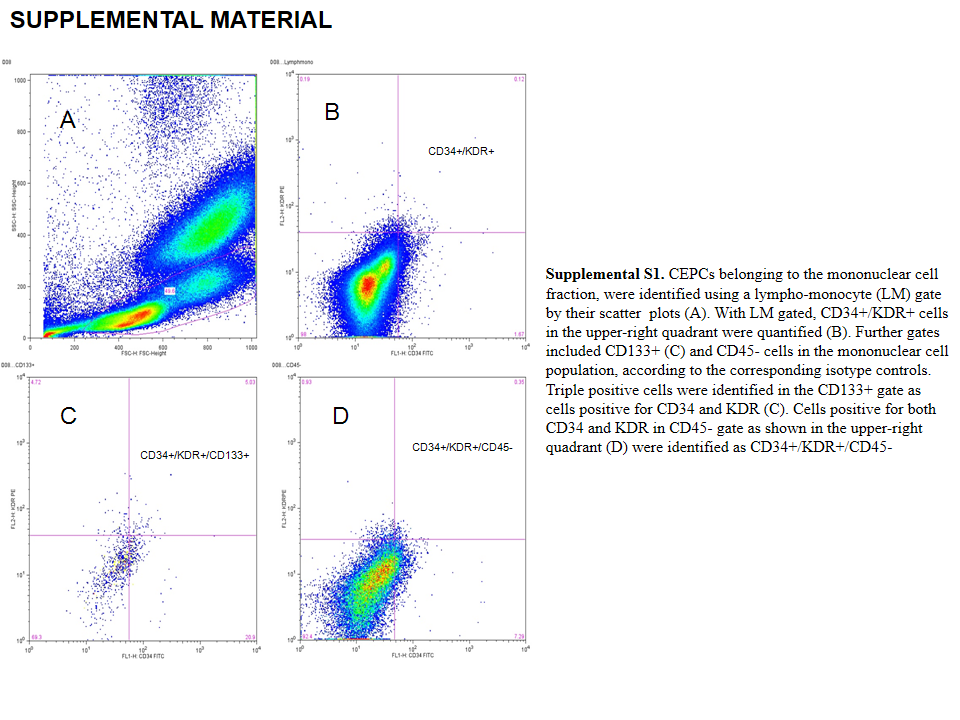

Supplement: Figure S1 — CEPCs belonging to the mononuclear cell fraction, were identified using a lympho-monocyte (LM) gate by their scatter plots (A). With LM gated, CD34+/KDR+ cells in the upper-right quadrant were quantified (B). Further gates included CD133+ (C) and CD45- cells in the mononuclear cell population, according to the corresponding isotype controls. Triple positive cells were identified in the CD133+ gate as cells positive for CD34 and KDR (C). Cells positive for both CD34 and KDR in CD45- gate as shown in the upper-right quadrant (D) were identified as CD34+/KDR+/CD45- . (PNG) [file pone.0083782.s001.png]

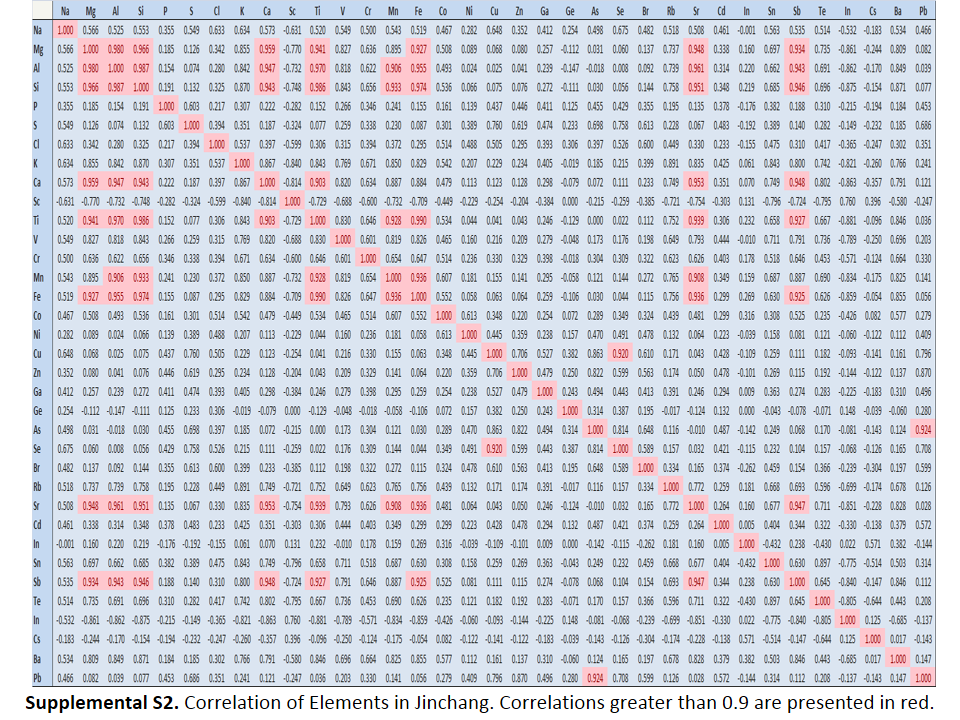

Supplement: Figure S2 — Correlation of Elements in Jinchang. Correlations greater than 0.9 are presented in red. (PNG) [file pone.0083782.s002.png]

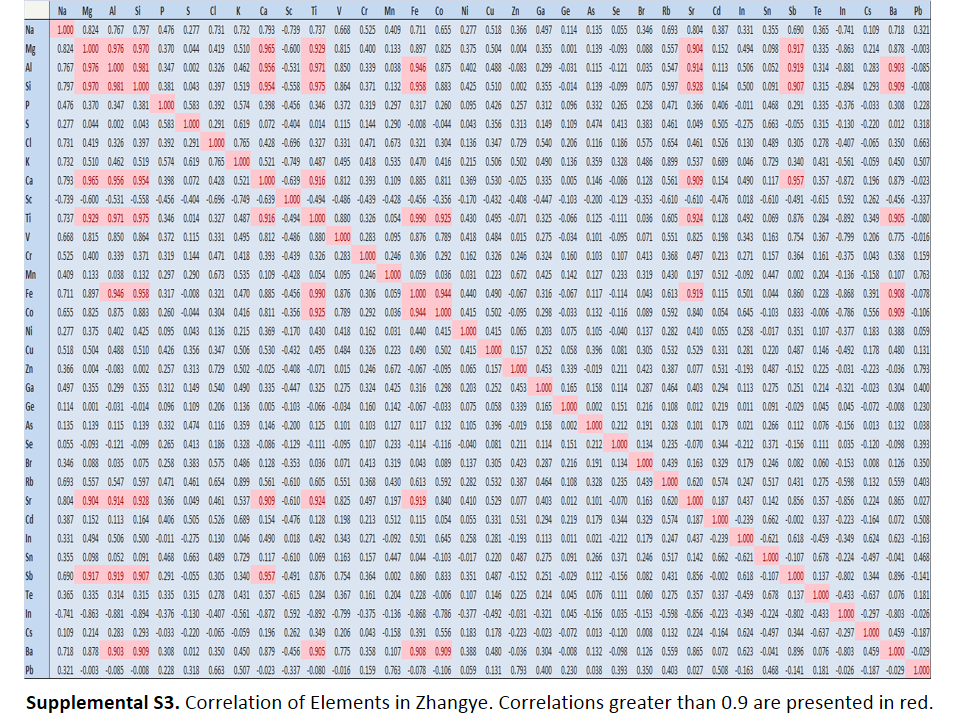

Supplement: Figure S3 — Correlation of Elements in Zhangye. Correlations greater than 0.9 are presented in red. (PNG) [file pone.0083782.s003.png]
